# Roll-Back Eradication of Bovine Tuberculosis (TB) From Wildlife in New Zealand: Concepts, Evolving Approaches, and Progress

**DOI:** 10.3389/fvets.2018.00277

**Published:** 2018-11-12

**Authors:** Graham Nugent, Andrew M. Gormley, Dean P. Anderson, Kevin Crews

**Affiliations:** ^1^Manaaki Whenua – Landcare Research, Lincoln, New Zealand; ^2^OSPRI, Christchurch, New Zealand

**Keywords:** bovine tuberculosis, eradication, TB, possums, disease freedom, wildlife surveillance

## Abstract

The New Zealand government and agricultural industries recently jointly adopted the goal of nationally eradicating bovine tuberculosis (TB) from livestock and wildlife reservoirs by 2055. Only Australia has eradicated TB from a wildlife maintenance host. Elsewhere the disease is often self-sustaining in a variety of wildlife hosts, usually making eradication an intractable problem. The New Zealand strategy for eradicating TB from wildlife is based on quantitative assessment using a Bayesian “Proof of Freedom” framework. This is used to assess the probability that TB has been locally eradicated from a given area. Here we describe the framework (the concepts, methods and tools used to assess TB freedom and how they are being applied and updated). We then summarize recent decision theory research aimed at optimizing the balance between the risk of falsely declaring areas free and the risk of overspending on disease management when the disease is already locally extinct. We explore potential new approaches for further optimizing the allocation of management resources, especially for places where existing methods are impractical or expensive, including using livestock as sentinels. We also describe how the progressive roll-back of locally eradicated areas scales up operationally and quantitatively to achieve and confirm eradication success over the entire country. Lastly, we review the progress made since the framework was first formally adopted in 2011. We conclude that eradication of TB from New Zealand is feasible, and that we are well on the way to achieving this outcome.

## Introduction

In 2016 the New Zealand government and agricultural industries jointly adopted the ambitious goal of nationally eradicating bovine tuberculosis (TB) from livestock and from all wildlife reservoirs by 2055 (1, 2). *Mycobacterium bovis*, the cause of TB, undoubtedly first arrived in New Zealand with imported cattle in the 1800s (3). By the mid-1900s it had spread into wildlife, and the disease became widely established in a highly susceptible and ubiquitous maintenance host, the introduced brushtail possum (*Trichosurus vulpecula*) (4), from which it often spills over to a number of other wildlife hosts, including feral pigs and wild deer (5) and feral ferrets (6).

Although diagnostic testing and removal of test-positive animals, coupled with slaughterhouse carcass inspection and livestock movement control to prevent further outbreaks, has reduced TB levels in livestock in many developed countries (7), the disease has been difficult to fully eradicate, especially in countries where TB is also independently cycling in wildlife reservoirs, such as badgers in Great Britain, wild boar and red deer in Spain, African buffalo and other species in South Africa, cervids (white tailed deer and elk) in North America, and brushtail possums in New Zealand (8). An exception is the successful eradication of TB from introduced water buffalo in Australia, where the “wildlife host” was a semi-domesticated or feral bovid with much the same TB epidemiological dynamics as cattle (9).

The main wildlife host in New Zealand (the brushtail possum) is very different from cattle: it is a small, nocturnal, and predominantly arboreal marsupial that is widespread and can occur at high densities (>20/ha) (4). Although it is a comparatively rapidly fatal disease for individuals, high-density possum populations can independently maintain TB (10) and can readily transmit TB to cattle (11). As a result of TB becoming widespread in possums in some parts of New Zealand in the 1970s, management of TB in New Zealand since then has therefore necessarily involved not only conventional management of the disease in livestock (12) but also efforts to break the TB cycle in possums though severe reductions in local possum density (“control”) (3). In this review we first very briefly summarize the c. 50-year history of TB management in New Zealand since it became both a livestock and wildlife problem, and then describe the key concepts and tools that have recently been developed to help achieve and confirm the new (2016) goal of national TB eradication.

We then focus more specifically on the concept of roll-back eradication. TB is established in wildlife in four main areas of New Zealand, which in total covered about 40% of the country in 2011. As the name implies, roll-back eradication entails locally eradicating TB from wildlife at the fringes of those four main areas and, over time, shrinking the size of each area from the outside in.

The key tool underpinning this concept is a Bayesian “Proof of Freedom” (PoF) framework, which is used to quantify the probability that TB is absent from possums in a specific area (P_free_). When that probability is considered high enough, an area is declared free of TB in wildlife and active management of TB in wildlife there ceases, with the management resources redirected to other areas where possums (and other wildlife) are still likely to be infected.

The PoF framework utilizes a number of information streams, including assessments of how effective efforts to reduce (control) possum densities have been, and infection surveillance data, not only from possums themselves but also from other TB hosts that can be infected by possums. We describe the background to the PoF framework (the concept of combining theoretical prediction of P_free_ with empirical TB-possum surveillance data), and how it was first implemented in 2011. We then summarize recent innovations, as follows:

- Simultaneous use of possum control efficacy data as well as TB surveillance data for updating the prior probability of freedom (13)
- Use of livestock as additional sources of data (sentinels) for detecting TB in wildlife (14)
- Use of decision theory to determine the optimal “stopping threshold” probability for declaring a particular local area free of TB (15)
- A description of how the progressive roll-back based on local areas can be scaled up to eventually confirm eradication success over the entire country (16).

Lastly, we review actual roll-back progress since 2011, and assess the likely accuracy of the P_free_ estimates given the lack (thus far) of any “post-freedom” failures (i.e., local re-emergence of TB in wildlife).

The review is based largely on the published work of the authors and our colleagues within Manaaki Whenua—Landcare Research, and builds on the comprehensive set of reviews about the epidemiology and management of TB in New Zealand wildlife in a 2015 special issue of the *New Zealand Veterinary Journal*. However, we also cite four reports documenting research that has not yet been published; these are available online via the DOIs appended to their citations.

We use “eradication” to refer to the complete or absolute absence of *M. bovis* from New Zealand livestock and wildlife, with negligible chance of re-invasion (except perhaps in human immigrants). Declaration of national eradication will signal the end of the programme. The term “TB freedom” is used in this paper specifically to denote a lesser but still high level of confidence that *M. bovis* is actually absent from wildlife in a given local area, either because wildlife there were never infected or because the disease has been eradicated. An area designated as free of TB can contain infected livestock if that infection is known to have not been caused by wildlife. The declarations of local-area freedom in wildlife therefore differ conceptually from the international standard for declaring national TB freedom in bovids and cervids, which explicitly permits a low level of continued infection in livestock (17). We also note that, for convenience, the term “disease” is used throughout this paper to encompass the presence of subclinical *M. bovis* infection as well as the presence of actual symptoms of disease (Appendix).

## Management of TB in new zealand

Since about 1995, management of TB in New Zealand has been conducted by a non-government agency (OSPRI, formerly TBFreeNZ, and even earlier the Animal Health Board). OSPRI represents a public–private partnership between government and the agricultural industries, and is responsible for implementing a formal National Pest Management Plan (NPMP) for TB (18). The initial NPMP in the mid-1990s aimed simply to try to prevent TB spreading further in wildlife. Then, in revisions in 2004 and 2011, it adopted more ambitious goals of not only reducing TB levels in livestock but also locally eradicating TB from possums and other wildlife (19, 18). By 2016 the national cattle herd TB annual period prevalence had been reduced to 0.09% (20), below the 0.2% threshold stipulated by the OIE (17) for declarations of whole-country TB freedom.

That success led to a fourth iteration of the NPMP, which adopted not only an ultimate goal of national eradication by 2055, but also intermediate goals of disease elimination from farmed livestock by 2026, and TB freedom in wildlife by 2040 (21). The long, 39-year timeline to eradication reflects the immensity of the problem: by 2004 TB was believed to be potentially established in wildlife in 10.5 million ha of New Zealand (c. 40% of the country), which encompassed not only farmed areas but also large tracts of remote, mountainous, and/or heavily forested lands, often occupied by high densities of possums (3). The scale of the problem was such that it was never economically feasible to immediately apply possum control over the whole of the affected area, so the eradication campaign has been, of necessity, centred on progressive reduction or “roll-back” of the areas thought to contain infected wildlife, termed vector risk areas (VRAs).

## The concept of roll-back eradication

The progressive roll-back concept is based on local TB management units within the VRAs, called vector control zones (VCZs), of which there were about 700 in 2011, with a typical size of 10,000–15,000 ha (but ranging from <1,000 ha to one of over 100,000 ha). The history of possum population control, livestock surveillance (herd test-and-cull and slaughterhouse inspection), and wildlife TB surveillance (necropsy) is recorded for each VCZ, and after 5–20 years of management an effort is made to quantitatively assess the probabilities that both livestock and wildlife are free of TB. When those probabilities are considered high enough, the VCZ is declared free of TB, and most of the management resources (funding) for that VCZ are then shifted to still-possibly-infected VCZs.

The broad theory and concepts underpinning this local PoF approach for wildlife are described in detail by Anderson et al. (22), but, briefly, are as follows.

- The effectiveness of possum control is assessed by field monitoring of possum relative abundance (or by inference from the known typical effectiveness of the control techniques)
- A spatially explicit model of TB dynamics in possums (23). is then used to predict the probability that TB could still be present given that level of control.
- Using Bayesian logic, this “prior” probability is then updated with empirical TB surveillance data. These data are based on necropsies of possums, or of spill-over hosts (such as pigs) that act as sentinels of TB in possums, to calculate a “posterior” probability of TB freedom P_free_ (22); that is, the probability of TB freedom given negative surveillance.
- Decisions on whether or not to declare the area free are then based on the estimated posterior probability.

The approach was first developed and used formally in 2011, and 174 VCZs totalling 2.05 million ha were declared free using this process in the subsequent 7 years (Crews, OSPRI, unpubl. data).

## The initial (2011) PoF framework for possums

### The TB freedom concept

The original concept underpinning the PoF framework for roll-back eradication (24) was simply that local management units (i.e., VCZs) can be quantitatively declared free of TB in possums (i.e., at some arbitrarily specified minimum level of confidence, usually, thus far, 95%) if:

- There is sufficient theoretical evidence (prediction) indicating that enough control has been applied to break the TB cycle in possums
- This prediction was backed up by empirical field surveillance data indicating a low probability of continued TB presence in possums.

For this, Bayes' rule was formulated as:

$$\begin{array}{l} P_{free}=\frac{Prior}{1-\left(SS\left(1-Prior\right)\right)} \end{array}$$

where P_free_ is the estimate of the “posterior” P_free_ required for decision-making, Prior is the measure of belief that an area is free of TB in wildlife based on historical control effort, and SS is a measure of surveillance sensitivity (formally defined below) describing how much effort has been made to find TB in possums without success. This simplified version of Bayes theorem assumes perfect specificity; i.e., surveillance is always negative when TB is not present in possums.

In operational terms, this usually translated into conducting intensive possum control for at least 5 (and often 10 or more) years and then implementing 2–3 years of field surveillance in an effort to detect any remaining TB in the residual possum population (25).

### Theoretical prediction of TB freedom based on control effectiveness

Because VCZs vary greatly in topography, habitat, possum density and TB history, the number of years of control (duration) and efficacy of control (percentage reduction in possum density) result in wide variation in control histories between VCZs, which is amplified by frequent changes in funding priorities. Prediction of whether a given control history is likely to have succeeded in eradicating TB is based on early modeling indicating TB has very little chance of persisting in possum populations that reduced to well below 40% of carrying capacity for 10–15 years (26). This was subsequently supported by field data (11), and a spatially explicit individual-based version of the Barlow model (the “SPM”) (23).

The SPM includes parameters representing both possum population dynamics (e.g., birth rates, mortality, density dependence, dispersal) and the epidemiological dynamics of TB in possums (e.g., transmission rates, TB-induced mortality). It is used within the PoF framework to simulate the effect of population control on reducing TB prevalence (23). To initialize these simulations, TB managers summarize the “control history” for the VCZ of interest, using (as far as possible) field measurements of the relative abundance of possums, most commonly a standardized index of trapping success (25). For un-monitored control operations, conservative estimates of control efficacy are assumed based on monitored outcomes at similarly managed sites. At least 100 simulations of the control history are then run with the SPM, with the prevalence of TB 30 years before the first control operation usually assumed to be 2.5% (based on the 2–5% prevalence typically recorded in unmanaged long-infected possum populations (4). The proportion of simulations in which TB is predicted to disappear is then used as a Bayesian “prior” P_free_ at the end of the series of control operations.

When the PoF framework was initially implemented, many of the VCZs being assessed had been under some form of possum control for more than two decades due to the strategic goal of the previous NPMP being one of ongoing TB suppression rather than eradication. When those long control histories were simulated in the SPM, the model would often predict eradication in every simulation (i.e., P_free_ = 1.0). As the predicted P_free_ exceeded the desired >95% minimum level of confidence, such VCZs could have been declared free on the basis of the model predictions alone, but TB managers required additional supporting empirical data from surveillance.

### Requirement for empirical possum-TB surveillance

The reason TB managers required additional information is that there is uncertainty about the accuracy of the SPM predictions. Not all SPM parameters have been formally validated, so it was accepted that some were likely to be wrong; for example, it was originally assumed that infected possums lived for about a year after becoming infected (23), but recent evidence indicates a much shorter duration of infection (27). Further, the accuracy of the control histories is often suspect as a result of data gaps. It was therefore decided by OSPRI that, as an operating principle, declarations of freedom would always require a minimum level of empirical post-control surveillance. To achieve this, a default maximum-permissible prior P_free_ of 0.9 was prescribed; in other words, if the SPM predicted (based on simulations of the duration and intensity of historical possum control) a prior of >0.90, it would be reduced to 0.90. In addition, at that time a posterior P_free_ of 0.95 was prescribed as the desired threshold (“stopping rule”) for declaring a VCZ free of possum TB. The gap between the maximum-permissible prior (≤0.90) and the stopping rule (0.95) meant that some surveillance was always needed.

The empirical TB surveillance required under this operating principle is obtained through necropsy surveys of possums or sentinel species. The surveys aim to quantify the surveillance sensitivity (SS), or the probability of detecting a TB-positive animal if the disease were actually present in a specified number of possums [the design prevalence, P^*^; (28)]. In principle, P^*^ should be set at one possum if the goal is confirming TB absence at the time of the survey. If the prior P_free_ is predicted to be at (or above) the maximum permitted level (0.95), and P^*^ = 1, then 53% of the possum populations would need to be tested (with perfect test sensitivity) to increase the posterior P_free_ to the 0.95 stopping rule for declaring local Tb freedom. More pragmatically P^*^ is now routinely set at 2, on the assumption that possum densities in the surveillance phase will almost always be well below the disease maintenance threshold, so TB is much more likely to die out rather than persist. That reduces the amount of field surveillance required by about 40%.

Once surveillance has been completed (and assuming no TB has been found in possums), the SPM-predicted prior P_free_ is updated annually using the surveillance sensitivity data obtained that year. If the posterior P_free_ exceeds the 0.95 stopping rule, the VCZ can be declared free of TB. If not, further surveillance is usually undertaken. However, in recognition that both the prior and the SS estimates are based on assumptions that may not all be valid, other qualitative factors (such as historical levels of infections, infection in neighboring VCZs, ease of remedying false declaration) are taken into consideration.

### Possum-TB surveillance in practice—alternative sampling units

(i) Possums as the sampling unit: The amount of surveillance required under the maximum-prior and stopping-rule settings above is large, usually equating to the equivalent of necropsying at least a third of the residual low-density possum population. Surveys of TB prevalence in possums had traditionally been conducted by capturing possums in leg-hold traps set for three or more nights, necropsying them, and conducting mycobacterial culture of tissues most likely to be infected, an approach believed to detect TB in about 95% of infected possums (29). Given negative surveillance (no TB detected), the SS could in theory then be calculated as a joint function of diagnostic test sensitivity and the proportion of the population sampled. However, the latter requires a precise estimate of local possum population size, which would be prohibitively expensive to routinely obtain. In addition, because surveys are usually conducted when possum densities are very low, much of the trapping effort results in empty traps. Such empty traps would not contribute to a conventional SS calculation based on number of possums necropsied, but failure to capture a possum at a particular site indicates a high probability that possums (and therefore TB) are absent from that site.
(ii) Traps and detection devices as the sampling unit: To circumvent the problem of not knowing possum population size, and to make use of the information provided by empty traps, a novel spatially explicit data-modeling approach to disease surveillance was developed (22), in which a VCZ is divided into 1 ha grid cells, and the cell rather than the individual possum is used as the sampling unit. Using data from all set traps (empty and captures), and estimated parameters for other studies on possum home range size and probabilities of trapping, this method estimates a VCZ-level SS (22).

To describe how this is done, assume that a trap is placed within the home range of a TB-infected possum, and that if that infected possum is captured it is necropsied and tested for TB. The probability of detecting TB given that TB is present (SS) is the product of (1) the probability of trapping the infected possum, and (2) the probability that the diagnostic test (mycobacterial culture) returns a positive result. By considering the trapping and diagnostics as two independent “tests” conducted in series, this allows us to include traps that do not capture possums (22, 30); i.e., the product can be applied to the trap whether or not it captures a possum, provided a diagnostic test is always performed whenever a possum is captured. This spatially explicit approach to estimating SS readily accounts for non-random sampling (so it does not require representative sampling).

A further extension of the ability to use empty traps (rather than possums) as sampling units involves the use of detection devices to reduce the trapping effort required. The detection devices [peanut-butter-lured chewcards (31)] are far lighter and easier to deploy than traps, and do not need to be checked daily, so they are used to cheaply identify the few small areas where possums are still present. Traps are then deployed only at those positive detection sites, and all possums captured are necropsied and tested for TB. The probability of detecting TB in this system (given TB presence) is the serial product of the probability of detection, the probability of capturing a possum in traps set at detection sites, and the probability of a positive diagnostic test. Although deploying traps only at detection sites results in a lower SS than if traps were deployed everywhere, the much lower cost of deploying chewcards and trapping only at detection sites makes this approach more cost effective, but still only affordable in readily accessible areas.

(iii) Spill-over hosts as the sampling unit: The high cost of direct possum surveillance led to the use of other spill-over host species as sentinels for TB presence in possums (32). By making data-based assumptions about sentinel home range size and the probability of a sentinel becoming infected when its home range overlaps with that of an infected possum, the surveillance sensitivity provided by these sentinels can also be estimated in a similarly spatially explicit way (22). Pigs, in particular, are highly sensitive sentinels because they very readily become infected in the presence of infected possums (33), have homes ranges that are much larger than those of possums (34), and survive in an infected state for far longer than possums (35). So where pigs can be readily obtained, surveying pigs can sometimes provide much cheaper possum-TB surveillance than would surveying possums themselves.

## Recent innovations

### Combining surveillance and final control

One shortcoming of the sequential “control-then-survey” approach outlined above is that it is only affordable in easily accessible farmland. There are many less accessible areas within VRAs where ground-based control and subsequent surveillance would be prohibitively expensive. Aerial poisoning provides an affordable alternative to ground control of possums in these areas (25), and sentinel pigs can sometimes provide the required level of surveillance at an affordable cost, but there are many areas where they do not.

A new approach for such difficult areas partially reverses the control-then-survey paradigm by conducting surveillance in conjunction with a final aerial control operation (13). That final operation will have been preceded by one or more earlier aerial poisoning operations, so the prior P_free_ (as predicted by the SPM) will already be high at the time of the final operation. A low level of direct possum TB surveillance is undertaken within a mark-recapture framework, involving trapping and marking (radio-collaring) and releasing possums just before the control operation and then, after the aerial poisoning, recapturing possums by searching for, recovering, and necropsying the killed possums.

**Figure 1:**
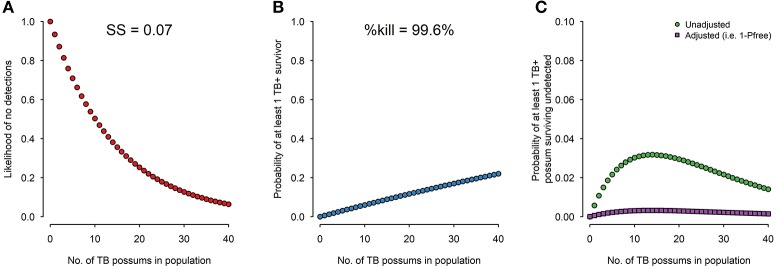
For the range of 0–40 TB possums in the population before surveillance and final control, **(A)** surveillance sensitivity (the likelihood of no detections) from a survey of 7% of the population with 95% diagnostic sensitivity; **(B)** TB survival probability (probability of at least one TB+ survivor) given a recorded control efficacy (% kill) of 99.6%; and **(C)** the probability of at least one TB possum surviving undetected which is shown both unadjusted (i.e., based solely on the evidence from the 2016/17 operation) and adjusted by the prior probability of freedom derived from the history of previous control, and is the complement of P_free_.

Provided no TB is detected, the likelihood of no TB being detected in the survey for each possible number of TB possums in the population (i.e., 0, 1, 2, 3,…, up to *N*: the pre-control population size) is calculated (Figure 1A). The efficacy of the control operation is determined from the percentage of radio-collared possums killed, and from that the probability that at least one TB possum would have survived if 0, 1, 2, 3,…, *N* infected possums were actually present (Figure 1B). The two probability distributions are then combined to estimate the probability that any infected possum could have survived undetected for each possible prevalence value (Figure 1C). Despite never knowing the number of TB possums in the population before surveillance and final control, we can use the maximum of the curve in Figure 1C, which corresponds to the worst-case scenario. The inverse of this can be further combined with the prior P_free_ to calculate the posterior P_free_.

The concept was successfully demonstrated in the Hauhungaroa Range in 2016/17 (13). This c. 80,000 ha area historically had some of the highest recorded levels of TB infection in wildlife, with almost all pigs and at least a third of the wild deer infected in the 1990s (4, 5). By 2016 all parts of the area had been under intensive control for 10–22 years, and the estimated P_free_ was 0.9. About 7% of the possum population (*N* = c. 4000) was necropsied, with no TB detected. Control efficacy was extremely high, with 99.6% of 241 radio-collared possums killed, resulting in a <4% probability that any infected possum would have survived undetected, which when combined with the prior P_free_ = 0.9 results in a posterior P_free_ >0.99 (Figure 1C).

The main advantage of this approach is the greatly reduced amount of surveillance needed, although that is partially offset by the need to obtain precise estimates of control efficacy (% kill) and the proportion of the population sampled. The other main advantage is that it enables faster declarations of freedom.

### Balancing control and surveillance effort and optimizing the stopping rule

The total costs of possum control and possum-TB surveillance depend on a number of factors (such as possum carrying capacity, ease of access, etc.), most of which have wide cost ranges. We modelled and compared management options to demonstrate that the optimal balance between the two activities necessary to achieve and verify eradication of TB from New Zealand wildlife varied greatly between VCZs (36). This work provided managers with a simple cost- and risk-evaluation framework they could use to identify the most expedient and economical ways of achieving and quantitatively verifying TB eradication from possums in a particular VCZ.

The initial stopping rule (posterior P_free_ > 0.95) was chosen subjectively by TB managers and their stakeholders (e.g., farming organizations, governmental funding bodies) to represent what they considered to be an “acceptable” level of risk of disease persistence. Our recent decision-theory modeling (15) indicates how the choice of stopping rule could be better optimized for each VCZ by explicitly including costs of surveillance and potential re-control costs.

If the posterior P_free_ are accurate, and if all VCZs are declared free as soon as they reach 0.95, it follows that 5% of VCZs will be falsely declared free of TB. TB managers therefore expect that in up to 5% of declared-free VCZs, TB will re-emerge in possums after possum control ceases, but will possibly not be detected for many years: where that occurs, potentially expensive re-control will obviously be required.

A higher stopping rule will result in a lower *expected cost* of re-control (the actual cost of re-control multiplied by the probability of incurring that cost). However, the cost of surveillance to achieve that higher target will increase. Conversely a lower stopping rule will result in a higher expected cost of re-control (due to an increased chance of incurring the actual re-control cost), but a lower surveillance cost due to stopping earlier. The optimal stopping rule for a VCZ will be the one that minimizes the total expected cost (expected costs of surveillance and re-control combined).

**Figure 2:**
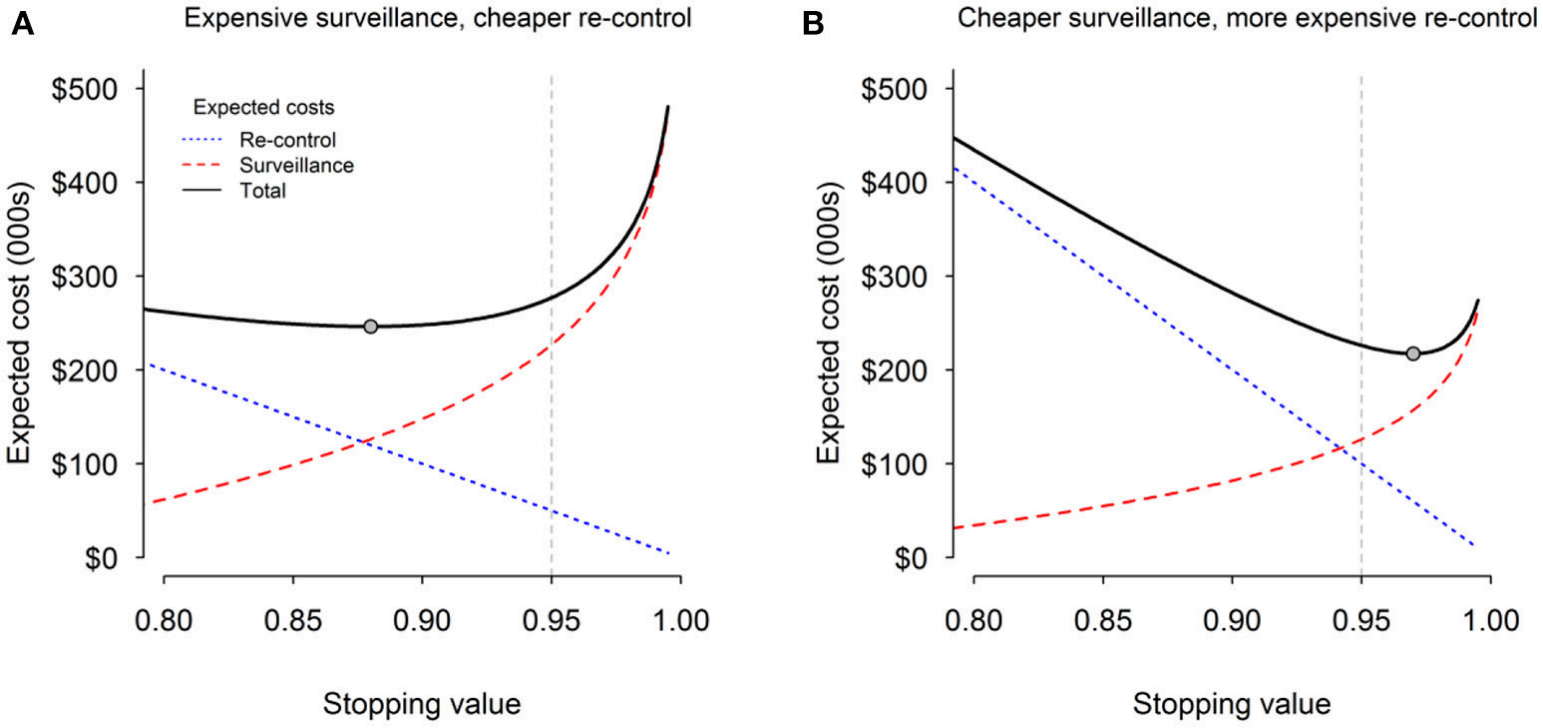
Cost of surveillance (red dashed line), expected cost of re-control (blue dotted line), and total combined expected cost (black solid line) for **(A)** expensive surveillance and cheaper re-control; and **(B)** cheaper surveillance and expensive re-control. The gray circles indicate the point associated with the minimum total expected cost. The vertical dashed gray line indicates the current default stopping threshold of 0.95.

Our analysis of the total expected costs indicates that where surveillance is relatively expensive compared with re-control, it will usually be more cost-effective to stop earlier than 0.95 at an increased risk of incorrect declaration [Figure 2A; (15)]. Conversely, where re-control is much more expensive than surveillance, it should be better to carry out more surveillance and choose a stopping threshold that is higher than 0.95 in order to mitigate the risk of incurring expensive re-control (Figure 2B).

This analysis has been used to develop a decision-support framework that provides guidance on how to optimize the economics of TB eradication, with the aim of eliminating the inefficiencies arising from relying on a single, predetermined, arbitrary stopping rule. Further work is now underway to see how best to include socio-political costs (the loss of credibility associated with incorrectly declaring an area free of TB), and therefore the risk profiles of decision-makers (risk averse vs. risk takers).

### Livestock as sentinels

Having expanded TB-possum surveillance options from surveying possums themselves to using data from traps and detection devices, and/or using spill-over hosts as possum-TB sentinels, we next explored the option of also using livestock as sentinels. Livestock are tested annually within all VCZs (and at longer intervals in areas designated as being free of TB in wildlife), and all livestock sent to slaughter are subject to rigorous inspection. The primary purpose of this testing and inspection is to determine TB levels in the livestock, but the same data can be used (at very little extra cost) to assess the likelihood TB is present in sympatric possums.

This might, at first sight, seem problematic because cattle are themselves maintenance hosts, so the occurrence of TB in a herd could be caused by recrudescence of latent in-herd infection or transfer of infection between herds by livestock movement rather than by transmission from wildlife. Identifying between-farms movement of livestock as the cause of a new outbreak in livestock (and therefore ruling out wildlife as the source) is facilitated by New Zealand's National Animal Identification and Tracing system, which is also managed by OSPRI. If, however, there is no detection of TB in livestock within a VCZ for many years, that obviously indicates there is no transmission from any source, including from possums.

We therefore developed an analytical technique to objectively use livestock as sentinels for TB in possums as an additional source of possum-TB surveillance information. For this, the spatially explicit modeling approach used to estimate SS from point-source data [i.e., the known kill locations of wildlife sentinels; (22)] was adapted to take into account the fact that the location of an individual cow or deer (and therefore the negative result of any TB test or slaughterhouse inspection) could not be localized to a single point. Instead, the surveillance data for the herd as a whole have to be spread evenly over the entire area in which they were grazed (14).

Because the probability of an individual cow becoming infected by a single infected possum somewhere on the same farm is believed to be very low (24), the SS provided by testing or inspection of a single cow is inevitably low. However, that poor individual sensitivity is offset by a large amount of livestock testing and slaughterhouse inspection data, available at very little cost because livestock are intensively surveyed annually within VRAs to confirm (or not) that the herds themselves remain free of TB (12). Thus, ongoing negative surveillance outcomes from livestock surveillance provide very-low-cost surveillance of TB in possums, reducing the amount of wildlife surveillance required.

Although not yet implemented, we envisage that in VCZs where herds were clear of TB before the end of the control phase, the livestock data will also be taken into account in identifying the prior P_free_. In addition, we believe the use of livestock as possum-TB sentinels will provide a crucial low-cost form of “post-freedom assurance surveillance,” particularly for on- and near-farm areas. The aim of such assurance surveillance is to provide the earliest possible detection of local eradication failure (i.e., persistence and re-emergence of TB in possums). It typically relies on passive (unfunded) rather than active (planned and funded) surveys. A key point is if TB does re-emerge, the numbers of infected possums will progressively increase over time, which will substantially increase the sensitivity of livestock surveillance in detecting the presence of TB.

### Scaling UP from VCZs to national eradication

To date, the roll-back eradication process has focused on achieving and declaring TB freedom at the VCZ level. This is done in a spatially strategic way to minimise the risk of reinvasion into VCZs previously declared free of TB. It may be tempting to simplistically conclude that the entire country will be free of TB once all VCZs have been declared free in this way. However, the declarations are probabilistic rather than certain. Given the 0.95 stopping rule used, there is a probability of up to 0.05 that the VCZ declared free was still infected. This error rate is compounded across all c. 800 VCZs so that the overall probability of total eradication from the country will be very close to zero (e.g., 0.95^800^ ≈ 0). This is not a bad result, because the bioeconomic optimisation modeling indicates that it is economically sensible to take some risks and be prepared to fail in some of the VCZs and have to re-initiate control and surveillance in them (Figure 2).

To account for this failure rate across VCZs in the context of the goal of declaring eradication from the entire country, the operational and decision processes can be divided into two stages (16). Stage I (“achieving freedom”) covers the initial efforts to eliminate TB from a given VCZ and the operational decision to declare that VCZ free of possum TB. Stage II (the “assurance” phase) requires (as noted above) ongoing but very-low-cost surveillance to either (i) quickly detect TB in cases where the declaration of freedom was false, or (ii) provide broad-scale SS data that can be used to calculate a probability of eradication at the level of whole regions, whole islands, or the whole country.

As outlined above, continued TB testing of livestock and slaughterhouse inspection is likely to provide such quantifiable assurance surveillance in on- and near-farm areas. Away from farmland there is currently mostly only limited passive and unquantified surveillance provided by recreational or commercial hunters, who might notice and report infection in any grossly infected pig or deer or ferret they kill, so consideration may need to be given to encouraging and quantifying the sensitivity of this kind of surveillance (or to funding low-intensity surveillance of sentinels and possums in high-risk areas with limited or no such passive surveillance).

Once all VCZs in a region, an island or the nation have been declared free, and TB is no longer being detected in them, the Stage II surveillance sensitivities across all VCZs will be aggregated to calculate a whole-area probability of eradication. Only when that exceeds some very high threshold (e.g., 0.99) will we be able to confidently declare that TB has been eradicated from New Zealand.

## Progress toward eradication

In the 7 years since the PoF framework was first formally adopted, 174 VCZs have been declared free, with all but 15 of those declarations based at least partly on the estimated posterior P_free_ (OSPRI, unpubl. data). A majority of these are farmed areas, and given the roll-back approach, most are at the former fringes of the VRAs where TB was generally not as well established in possums as in more central parts of VRAs. Nonetheless, the total does include several of the worst-affected forest areas in which TB was long established in wildlife at high levels, including the Hauhungaroa Range mentioned above. In total, over 2.05 million ha has now been declared free using the PoF framework, about 20% of the total area designated VRA in 2011.

By 2018 these 174 VCZs had been free for an average of 3.8 years, equating to 694 years of VCZ freedom. If TB was still present in a declared-free VCZ, we expect that it would re-emerge and be detected (on average) within 4–5 years of being declared free (at least where high numbers of cattle are TB tested and/or slaughtered annually). If so, and if up to 5% of declarations were false, we would have expected the detection of re-emergent TB in 5–10 VCZs by now. There have been none (OSPRI, unpubl. data).

**Figure 3:**
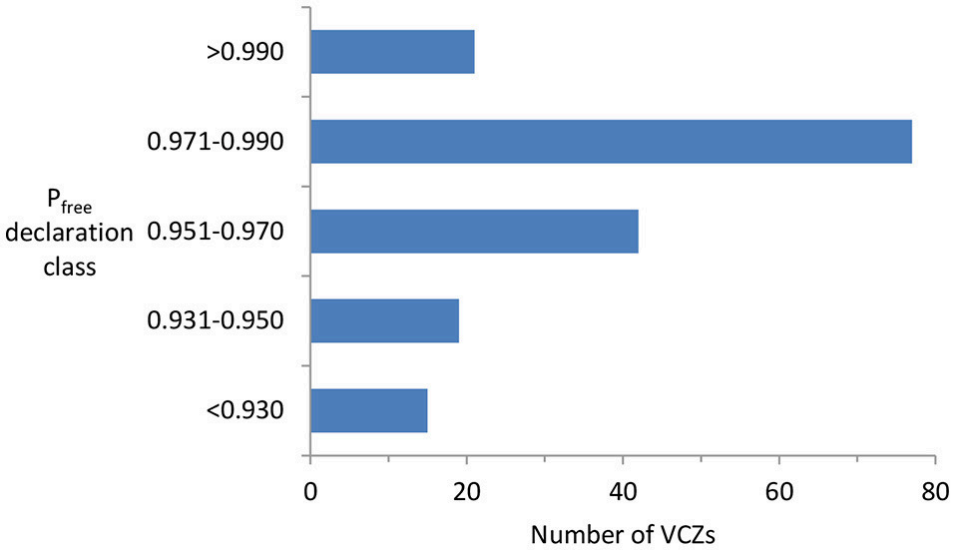
Frequency distribution of the number of VCZs declared free between 2011 and 2018 in relation to the Pfree estimates (calculated using a design prevalence of 2, and grouped into five classes) at the time of declaration. The “Pfree declaration class <0.930” represents VCZs declared free on a largely qualitative basis rather than a quantitative one.

The lower-than-expected failure rate partly reflects the fact that many of the VCZs were not declared free until the posterior P_free_ was substantially above the 0.95 stopping rule (Figure 3). This is largely because until recently the PoF process was very largely retrospective: control and surveillance were conducted according to a fixed standard schedule (25), and only on completion of that were the data analyzed. That resulted, in many instances, in far more surveillance being done than was strictly necessary. To help avoid that in future, we have developed an online decision-support tool (https://landcare.shinyapps.io/JESS), which enables managers to determine, for any given prior P_free_, the minimum amount of surveillance needed to reach the stopping rule.

A second possible reason for the low failure rate is the conservative setting, by OSPRI, of a maximum prior P_free_ of 0.90 even when the SPM predicts that a given control history would have eradicated TB in 100% of simulations. If the model predictions were accepted as accurate, the posterior P_free_ estimates would have been higher (and therefore the expected failure rate lower).

Another possible reason for the low failure rate is that the SPM may be predicting that eradicating TB from possums is more difficult than it actually is, biasing the prior P_free_ estimates low. A converse point is that the low failure rate provides some validation of the SPM predictions: if the SPM was falsely providing overly optimistic predictions of the probability of eradication, there would have been more failures observed than expected.

There are some indications that the SPM was indeed biased when applied to areas in which possum carrying capacity was well below average. In such areas the SPM often predicted TB would not persist even without control, despite evidence that TB had actually been detected in possums in some such areas (Crews, OSPRI, unpubl. data). To remedy that mismatch between model prediction and reality, the SPM has been revised by changing the possum–possum contact-rate function from one based on distance between home range centres to a more realistic one based on home range overlap (37), resulting in a greater amount of control being needed than previously for the model to predict eradication in areas of poor possum habitat.

Whatever the reason, there is evidence that the prior P_free_ estimates being used are conservative. In 2015, key TB managers were asked to subjectively assign prior P_free_ estimates for all VCZs in which TB surveys of possums had recently been conducted (38). There were 133 surveys in VCZs that had been under possum control for many years and that had prior P_free_ estimates in the range 0.70–0.95. Using conventional probability theory, the surveillance sensitivity estimates from these surveys were used to determine the probability that those surveys would have detected TB if it were actually present as frequently as the managers' prior P_free_ suggested it should be. Collectively, these 133 surveys should have resulted in 13 detections of infected possums if the P_free_ estimates were accurate, but again there were no detections. The implication is that there was far less infection in long-managed populations than managers believed, based on their experience with the PoF framework.

## Discussion

The evolution of the TB eradication programme in New Zealand (3) is the product of an adaptive management (39) effort in which management decisions are evidence based, and new research questions and developments are shaped by management outcomes and needs. New Zealand's TB management agency, OSPRI, has historically funded, and continues to fund, robust and innovative science to support their desire for evidence-based decision-making. There is a strong focus on continual improvement, with a constant appetite for exploring new methodologies in order to achieve TB freedom ever more cost-effectively and ever more quickly.

A key factor in the continued success of the components of the “TBfree” programme that are specifically aimed at eliminating TB in wildlife has been the strong partnership and close working relationships over more than two decades between the end-user (OSPRI) and researchers at New Zealand's main terrestrial environment research institute (Manaaki Whenua—Landcare Research). This relationship, and the research findings that have flowed from it, has resulted in wide-ranging changes in operational strategies and activities. In particular, the PoF framework has become an integral part of TB management, with the posterior P_free_ increasingly recognized as the ultimate management performance metric. We believe that the challenges and successes of this collaborative experience will be instructive for other countries aiming to manage or eradicate TB from very large areas.

With less than a quarter of the area believed to contain infected wildlife declared free so far, there is clearly still an immense amount of management (and research) to be done. However, the success and progress to date, as well as the development and implementation of new methodologies and smarter decision-making tools, means that New Zealand is well on the way to eliminating TB in both livestock and wildlife, and is well on track to achieve the goal of disease eradication by 2055.

## Author contributions

GN, AG, and DA reviewed and summarized their own bodies of work and collectively integrated those summaries into the overall review. KC (and other OSPRI staff) provided data on eradication progress, and also reviewed the whole document.

### Conflict of interest statement

Most of the research and development described in detail here was conducted the Manaaki Whenua—Landcare Research authors in collaboration with, and with part or full funding from OSPRI, New Zealand's TB management agency, of which author KC is Head of Programme (Disease Management). The remaining authors declare that the research was conducted in the absence of any commercial or financial relationships that could be construed as a potential conflict of interest.

## Appendix: list of abbreviations and definitions used in the text

**Table:**

Assurance surveillance	Possum-TB surveillance undertaken after an area has been declared free, usually using unplanned, low-cost, “passive” methods (hunter observations from possums, deer and pigs, and livestock testing or slaughterhouse inspection data collected for other purposes).
Control	Reduction in possum density by lethal trapping or poisoning.
Control	efficacy Effectiveness of possum population reduction (% kill).
Control history	Summary of the duration (span of years) and intensity (control effectiveness or efficacy).
Disease	A term of convenience used to encompass the presence of subclinical *M. bovis* infection as well as the presence of actual symptoms of disease.
Eradication	Complete or absolute removal of *M. bovis* infection from all animals in an area with negligible chance of re-establishment.
Freedom	High but not absolute probability of absence of *M. bovis* infection from all animals in a specified area at a specific time.
Max prior	Maximum permissible prior: a subjective precautionary prescription of the maximum value that can be ascribed to the prior (defined below).
NPMP	National Pest Management Plan: a national plan required under New Zealand biosecurity legislation, first developed in the mid-1990s and revised and updated in 2005, 2011, and 2016.
P_free_	Probability of absence of *M. bovis* infection from possums in a specified area at a specific time.
PoF	Proof of Freedom: a Bayesian belief-updating framework in which a quantitative estimate of the belief (confidence) that an area is free of TB at a given time (the “prior”) is updated at a later time by the new information gathered between the two times to produce a new estimate (the posterior). The updating takes into account the possibility of re-introduction of new infection.
Posterior	A quantitative probabilistic estimate of the belief (confidence) that an area is free of TB at a given time that is derived by updating an initial prior belief with new empirical evidence of TB absence.
Prior	A quantitative probabilistic estimate of the belief (confidence) that an area is free of TB at a given time
P^*^	Design prevalence: the specified surveillance target.
Re-control	Additional control required when an area is falsely declared free of TB, resulting in eventual to re-emergence of the disease and a need to again reduce possum densities in a further effort to break the TB cycle in possums.
Sentinels	Spill-over hosts of TB that can become infected by transmission from possums, but which do not independently maintain the infection, either because they are largely end hosts (pigs, deer, and ferrets in most places), or because they are subject to effective TB management (livestock).
SPM	Spatial Possum Model: an individual-based, spatially explicit simulation model of the eco-epidemiological dynamic of TB in possums, which is used to predict the likely effect of historical possum control on TB prevalence in possums.
SS	Surveillance sensitivity: the probability of finding an *M. bovis* infected animal in a particular survey sample of possums or sentinels if *n* TB possums were actually present in the area surveyed, with *n*/*N* (the population size) being the design prevalence P^*^.
Stopping rule	The desired or prescribed level of confidence required before an area can be declared free of wildlife TB.
Surveillance	Empirical survey of animal disease status (through necropsy and mycobacterial culture of wild animals, or TB testing and/or slaughterhouse inspection of livestock).
TB	Bovine tuberculosis, caused by infection with *Mycobacterium bovis*.
TB possum	A possum with *M. bovis infection*.
VCZ	Vector control zones: formally defined areas, typically of 10,000–20,000 ha, used for planning possum control and surveillance, and forming the primary spatial management unit.
VRA	Vector risk area: an area considered to have a non-zero probability of containing infected wildlife (see https://ospri.co.nz/our-programmes/tbfree/about-the-tbfree-programme/wildlife-and-pest-management/vector-risk-areas/). “Vector free” areas (all of the non-VRA land) can contain infected livestock provided there is high confidence that the infection originated in other livestock elsewhere.
